# SARS-CoV-2 Serology and Self-Reported Infection Among Adults — National Health and Nutrition Examination Survey, United States, August 2021–May 2022

**DOI:** 10.15585/mmwr.mm7148a4

**Published:** 2022-12-02

**Authors:** Lara J. Akinbami, Deanna Kruszon-Moran, Chia-Yih Wang, Renee J. Storandt, Jason Clark, Minsun K. Riddles, Leyla K. Mohadjer

**Affiliations:** ^1^Division of Health and Nutrition Examination Surveys, National Center for Health Statistics, CDC; ^2^Westat Corporation, Rockville, Maryland.

CDC COVID-19 surveillance systems monitor SARS-CoV-2 antibody prevalence to collect information about asymptomatic, undiagnosed, and unreported disease using national convenience samples of blood donor data from commercial laboratories (*1*,*2*). However, nonrandom sampling of data from these systems could affect prevalence estimates (*1*–*3*). The National Health and Nutrition Examination Survey (NHANES) collects SARS-CoV-2 serology data among a sample of the general U.S. civilian population (*4*). In addition, NHANES collects self-reported COVID-19 vaccination and disease history, and its statistical sampling design is not based on health care access or blood donation. Therefore, NHANES data can be used to better quantify asymptomatic SARS-CoV-2 infection prevalence and seropositivity attained through infection without vaccination. Preliminary NHANES 2021–2022 results indicated that 41.6% of adults aged ≥18 years had serology indicative of past infection and that 43.7% of these adults, including 57.1% of non-Hispanic Black or African American (Black) adults, reported never having had COVID-19, possibly representing asymptomatic infection. In addition, 25.5% of adults whose serology indicated past infection reported never having received COVID-19 vaccination. Prevalences of seropositivity in the absence of vaccination were higher among younger adults and Black adults, reflecting the lower observed vaccination rates among these groups (*5*). These findings raise health equity concerns given the disparities observed in SARS-CoV-2 infection and COVID-19 vaccination. Results from NHANES 2021–2022 can guide ongoing efforts to achieve vaccine equity in COVID-19 primary vaccination series and booster dose coverage.*

The 2-year sample design of NHANES 2021–2022, includes 30 primary sampling units (usually a county) that are visited sequentially. In each 12-month data collection period, a nationally representative sample of 15 primary sampling units are visited. Preliminary data for adults aged ≥18 years from the first 10 primary sampling units (visited during periods of SARS-CoV-2 Delta [August–November 2021] and Omicron [December 2021–May 2022] variant predominance) (*6*) were analyzed as a convenience sample because data for all 15 primary sampling units were not yet available.^†^ Analysis of preliminary unweighted NHANES data was conducted to examine SARS-CoV-2 antibody status in association with demographic characteristics and self-report of ever having had COVID-19 illness and having received ≥1 dose of COVID-19 vaccine. Sera were tested for anti-spike (anti-S) antibodies (which are produced in response to COVID-19 vaccination, SARS-CoV-2 infection, or both) using the Ortho VITROS Immunodiagnostic Products Anti-SARS-CoV-2 Total Reagent Pack.^§^ Anti-nucleocapsid (anti-N) antibodies, which are produced only in response to SARS-CoV-2 infection, were assessed with the Total N Antibody Reagent Pack.^¶^ Seroprevalence was calculated by age, sex, race and Hispanic origin, and education in persons with combined anti-S–positive and anti-N–positive test results (infected, possibly vaccinated) and those with combined anti-S–positive and anti-N–negative test results (vaccinated, not infected). Among 1,581 participants with serology results, seven were excluded (including three with “don’t know” or “refused” responses for self-reported COVID-19 history and four with a combined serology result of anti-S–negative and anti-N–positive**) leaving an analytic sample of 1,574. Analyses were performed using SAS software (version 9.4; SAS Institute). Final survey weights were unavailable at the time of this report because they are not calculated until the conclusion of the 2-year data collection cycle. Because NHANES uses a complex sampling design, simple random sampling assumptions for statistical testing are not appropriate. Therefore, statistical comparisons were not performed and references to differences among groups are based on observation only. The NHANES protocol was approved by the National Center for Health Statistics Ethics Review Board and was conducted consistent with applicable federal law and CDC policy.^††^

During August 2021–May 2022, a total of 91.5% of adults included in NHANES had SARS-CoV-2 anti-S antibodies and 41.6% had anti-N antibodies. The percentage of adults with anti-S–positive, anti-N–positive serology (infected, possibly vaccinated) (Figure 1) was 41.6% overall and declined with age (59.7% among adults aged 18–29 years versus 30.2% among those aged ≥70 years); anti-S–positive, anti-N–positive prevalences were equivalent to anti-N–positive prevalences. The percentage of adults with this serologic profile also varied by race and Hispanic origin; 59.2% Hispanic, 45.9% Black, and 30.6% non-Hispanic White (White) adults were infected and possibly vaccinated. Percentages also declined with increasing education level, with 49.0% adults with less than high school education versus 37.5% of those with at least some college being infected and possibly vaccinated. In contrast, the percentage of adults with anti-S–positive, anti-N–negative results (vaccinated, not infected) (Figure 1) was 49.9% overall, increased with age (28.1% among adults aged 18–29 years versus 64.7% among those aged ≥70 years), was lower among Hispanic (35.3%) and Black adults (46.7%) and higher in White adults (58.9%), and lower in adults with less than high school education (42.5%) and higher in those with at least some college (55.4%).

**FIGURE 1:**
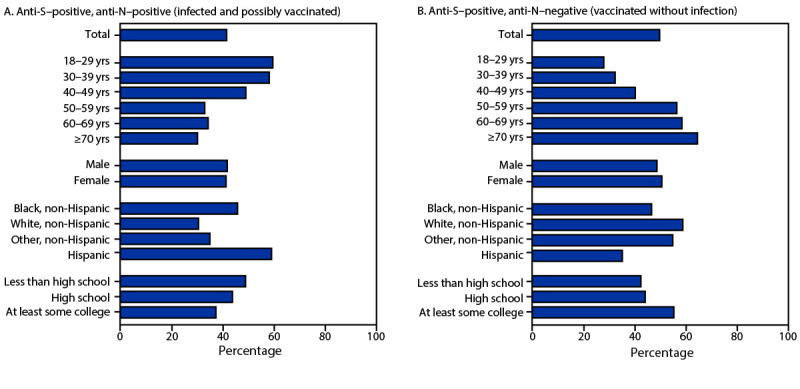
Combined SARS-CoV-2 anti-spike* and anti-nucleocapsid^†^ antibody testing results among adults aged ≥18 years who were infected and possibly vaccinated (A) and those vaccinated without infection (B), by age group, sex, race and Hispanic origin,^§^ and education — National Health and Nutrition Examination Survey, United States, August 2021–May 2022^¶^ **Abbreviations**: anti-N = anti-nucleocapsid; anti-S = anti-spike. * Positivity for SARS-CoV-2 anti-S antibodies (previous infection, vaccination, or both). ^†^ Positivity for SARS-CoV-2 anti-N antibodies (previous infection). ^§^ The category “other, non-Hispanic” includes non-Hispanic participants who reported being either American Indian or Alaska Native, Asian, Native Hawaiian or other Pacific Islander, or multiple race. ^¶^ Preliminary sample = 1,574, unweighted data; information on education was missing for 63 adults.

Among 655 adult participants with anti-S–positive, anti-N–positive serology results (indicating infection), 43.7% reported that they had never had COVID-19 (Figure 2). This percentage was higher among Black adults (57.1%) and adults with less than high school education (57.8%) than among adults of other racial and ethnic groups and among those with higher educational attainment. Among anti-S-positive, anti-N-positive adults, 25.5% reported never having received any COVID-19 vaccination (Figure 2). Percentages of respondents who reported not having been vaccinated decreased with age (31.6% among adults aged 18–29 years versus 18.8% among adults aged ≥70 years). A higher percentage of Black adults (31.3%) and a lower percentage of Hispanic adults (21.4%) with serologic evidence of infection reported never having received COVID-19 vaccination.

**FIGURE 2:**
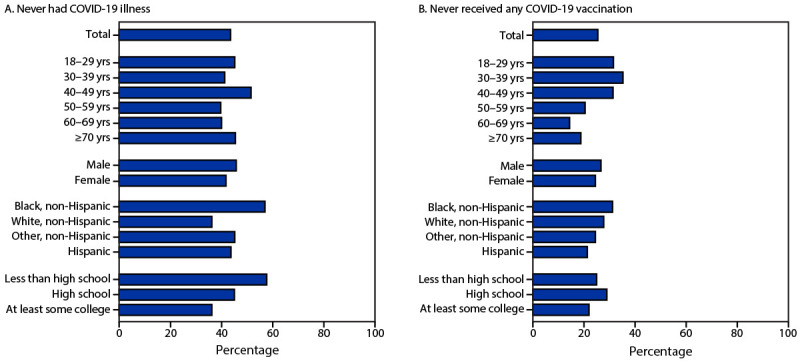
Percentage of adults aged ≥18 years with both SARS-CoV-2 anti-spike* and anti-nucleocapsid^†^ antibodies who reported never having had COVID-19 (A)^§^ or never having received any COVID-19 vaccine (B),^¶^ by age group, sex, race and Hispanic origin,** and education — National Health and Nutrition Examination Survey, United States, August 2021–May 2022^††^ * Positivity for SARS-CoV-2 anti-spike antibodies (previous infection, vaccination, or both). ^†^ Positivity for SARS-CoV-2 anti- nucleocapsid antibodies (previous infection). ^§^ Negative response to the question, “Have you ever had COVID-19, or the illness caused by the Coronavirus Disease 2019?” ^¶^ Responded “zero doses” to the question, “How many doses of COVID-19 vaccine have you received? Please include booster shots and any additional doses.” ** The category “other, non-Hispanic” includes non-Hispanic participants who reported being either American Indian or Alaska Native, Asian, Native Hawaiian or other Pacific Islander, or multiple race. ^††^ Preliminary sample = 655, unweighted data; information on education was missing for 36 adults.

## Discussion

Preliminary analyses of unweighted NHANES data during August 2021–May 2022, found that 41.6% of adults had SARS-CoV-2 anti-N antibodies, consistent with previous infection. CDC’s nationwide commercial laboratory surveillance system estimated a higher anti-N seroprevalence (57.7%) among persons of all ages for the period January–February 2022 (*2*). This difference was not unexpected, given that the commercial laboratory estimate included sampling only after the more infectious SARS-CoV-2 Omicron wave (*6*) and included children, whose seroprevalence is higher than that of adults (*2*). However, patterns by age group and sex were similar between NHANES and commercial laboratory data sources, with declining anti-N antibody prevalence associated with increasing age and similar prevalences among males and females. Similar to the patterns in anti-N antibody seroprevalence by race and Hispanic origin observed in NHANES, CDC national blood donor surveillance data for persons aged ≥16 years through December 2021 also found higher anti-N seroprevalence in persons belonging to racial and ethnic minority groups (*1*,*2*). Antibody patterns in seropositive racial and ethnic minority adults were less likely to be consistent with vaccination and more likely to suggest past infection than those observed in seropositive White adults. These patterns are consistent with survey data indicating lower vaccination coverage and higher infection rates among Hispanic and Black adults than among White adults (*5*,*7*,*8*).

These findings confirm many patterns observed in other seroprevalence studies based on convenience samples that reflect increased vaccination rates among older persons and higher infection rates among younger persons (*2*). Currently, few U.S. data sources can provide data on antibody status and self-reported COVID-19 illness and vaccination. Preliminary NHANES data indicated that 43.7% of adults with serologic evidence of SARS-CoV-2 infection reported never having had COVID-19 and approximately one half of Black adults and those with lower educational attainment were possibly asymptomatically infected. Younger adults and Black adults with unidentified infections might have been more likely to lack access to testing and to have unknowingly exposed others, resulting in disparities in community transmission. In this way, undiagnosed infections could have amplified disparities in infection rates and outcomes (*2*,*3*). Furthermore, estimates of infection based on antigen testing results are likely underestimated. In addition, among anti-S–positive, anti-N–positive (infected and possibly vaccinated) adults, a higher percentage of younger and Black adults did not report any COVID-19 vaccination, suggesting that higher percentages of these groups acquired antibodies through infection rather than vaccination. Conversely, the antibody pattern consistent with vaccination without infection (anti-S–positive, anti-N–negative) was lower among Hispanic and Black adults and those with less than high school education.

The findings in this report are subject to at least five limitations. First, self-reported COVID-19 vaccination and infection history could be subject to social desirability bias. Second, to provide preliminary results, data from the first 10 primary sampling units were analyzed before completion of the 2-year NHANES data collection cycle. Because final survey weights were unavailable, no adjustment was made for nonresponse and unequal probability of selection by age. In addition, the unweighted sample is subject to bias and does not represent a particular population. For example, the population aged ≥60 years is overrepresented in this sample. Third, among the 10 primary sampling units included, those visited earlier in the survey cycle, during the predominance of the Delta variant, are combined with those visited later during the Omicron-predominant period. Thus, the seroprevalence estimates during these two variant periods are averaged over the period represented by NHANES data. Fourth, the observed seroprevalence in these 10 primary sampling units might differ from that in the primary sampling units that were not yet visited. Finally, seroprevalence might further underestimate the cumulative number of vaccinations and infections: some persons with infection or vaccination might remain seronegative (*9*), and infection after vaccination might result in lower anti-N titers (*10*).

CDC recommends that everyone remain up-to-date with COVID-19 vaccination. Consistent with findings from other seroprevalence studies, preliminary NHANES 2021–2022 results raise health equity concerns given the disparities observed in SARS-CoV-2 infection and COVID-19 vaccination. These results can guide ongoing efforts to achieve vaccine equity in COVID-19 primary vaccination series and booster dose coverage.

**Summary**

**What is already known about this topic?**

A high percentage of U.S. adults have antibodies to SARS-CoV-2, attained through vaccination, infection, or both.

**What is added by this report?**

During August 2021–May 2022, 41.6% of a convenience sample of adults had both anti-spike antibodies (indicating previous infection or vaccination) and anti-nucleocapsid antibodies (indicating previous infection only); 43.7% of these persons were possibly asymptomatically infected. Prevalence of serologic patterns consistent with vaccination without infection was lower among adults who were younger, Hispanic and non-Hispanic Black or African American adults, and persons with less education.

**What are the implications for public health practice?**

CDC recommends that everyone stay up to date with COVID-19 vaccination. These results can guide ongoing efforts that are needed to achieve equity in primary series vaccination and booster dose coverage.

## Footnotes

* https://www.cdc.gov/coronavirus/2019-ncov/vaccines/stay-up-to-date.html (Accessed September 12, 2022).

† Public release of the full data set for 30 primary sampling units for the 2021–2022 NHANES cycle on the NHANES website occurs upon completion of data collection and processing. The 10 primary sampling unit data set used for this analysis is available in the National Center for Health Statistics Research Data Center. The locations of the primary sampling units included in NHANES are never publicly released to protect respondent confidentiality. https://www.cdc.gov/nchs/nhanes/participant/participant-confidentiality.htm

§ https://www.fda.gov/media/136967/download (Accessed September 5, 2022).

¶ https://www.fda.gov/media/151027/download (Accessed September 5, 2022).

** This pattern might reflect more recent infection given that anti-S antibody levels might rise more slowly than anti-N antibody levels after infection. However, these participants were still excluded for clarity of presentation. https://www.frontiersin.org/articles/10.3389/fmicb.2020.584251/full

†† 45 C.F.R. part 46, 21 C.F.R. part 56; 42 U.S.C. Sect. 241(d); 5 U.S.C. Sect. 552a; 44 U.S.C. Sect. 3501 et seq.
